# Simulation of Cone-Jet and Micro-Drip Regimes and Printing of Micro-Scale Patterns on PET Substrate

**DOI:** 10.3390/polym14132683

**Published:** 2022-06-30

**Authors:** Dazhi Wang, Zeshan Abbas, Liangkun Lu, Shiwen Liang, Xiangyu Zhao, Pengfei Xu, Kuipeng Zhao, Liujia Suo, Yan Cui, Penghe Yin, Bin Tang, Jin Xie, Yong Yang, Junsheng Liang

**Affiliations:** 1 Key Laboratory for Micro/Nano Technology and System of Liaoning Province, Dalian University of Technology, Dalian 116024, China; hopenotout1214@mail.dlut.edu.cn (Z.A.); llk@mail.dlut.edu.cn (L.L.); zxy4195@dlut.edu.cn (X.Z.); xupfupc@163.com (P.X.); kuipengzhao@mail.dlut.edu.cn (K.Z.); suolj@dlut.edu.cn (L.S.); yanc@dlut.edu.cn (Y.C.); phyin@dlut.edu.cn (P.Y.); engineeringjsliang@dlut.edu.cn (J.L.); 2 Ningbo Institute, Dalian University of Technology, Ningbo 315000, China; liangsw_nbi@dlut.edu.cn; 3 Key Laboratory for Precision and Non-Traditional Machining Technology of Ministry of Education, Dalian University of Technology, Dalian 116024, China; 4 Laboratory of Precision Manufacturing Technology, CAEP, Mianyang 621900, China; john46311@hotmail.com (B.T.); xiejin_nwpu@163.com (J.X.); 5 Ningbo Yongxin Optics Co., Ltd., Ningbo 315000, China; yy@yxopt.com

**Keywords:** MEMS devices, PET substrate, direct writing method, drop-on-demand, micro-patterns

## Abstract

The fabrication of various micro-patterns on polymer insulating substrates is a current requirement in micro-electromechanical system (MEMS) and packaging sectors. In this paper, we use electrohydrodynamic jet (E-Jet) printing to create multifaceted and stable micro-patterns on a polyethylene terephthalate (PET) substrate. Initially, simulation was performed to investigate optimized printing settings in phase field physics for the usage of two distinct functional inks. A series of simulation experiments was conducted, and it was determined that the following parameters are optimised: applied pressure of 40 kPa, high pulse voltage of 1.95 kV, low dc voltage of 1.60 kV, duty cycle of 80%, pulse frequency of 60 Hz, printing height of 0.25 mm, and printing speed of 1 mm/s. Then, experiments showed that adjusting a pressure value of 40 kPa and regulating the SEMICOSIL988/1 K ink to print micro-drops on a polymer substrate with a thickness of 1 mm prevents coffee staining. The smallest measured droplet size was 200 μm. Furthermore, underfill (UF 3808) ink was driven with applied pressure to 50 kPa while other parameters were left constant, and the minimum size of linear patterns was printed to 105 μm on 0.5-mm-thick PET substrate. During the micro-drip and cone-jet regimes, the consistency and diameter of printed micro-structures were accurately regulated at a pulse frequency of 60 Hz and a duty cycle of 80%.

## 1. Introduction

The fabrication of different micro/nano-structures on polymer insulating substrates using E-Jet printing technology has generated widespread interest due to its extensive applications in flexible electronic devices [1,2], the biomedical sector [3], optoelectronic fields [4] and other different disciplines. The use of E-Jet printing technology for micro/nano-patterns have many advantages compared to traditional photolithography and ink-jet printing, i.e., a three-dimensional structuring [5], a high-resolution patterning technique [6], high efficiency of resource dissipation [7], high-speed printing with nano-sized structures [8], and a contactless method [9].

The E-Jet printing technology is usually categorized into direct writing and drop-on-demand type, depending on the mechanisms used to generate continuous-line structures and micro-droplets [10,11]. The technology has recently attracted attention for printing micro/nano-sized patterns because it can print much smaller micro-structures than the needle diameters [12]. Furthermore, many versatile materials, such as metal, organic, and even biological material, can be used without thermal damage. The needle structure used in this technology is simpler than that used in other types. In the E-Jet method, the charged fluid is extracted from the needle tip when the electrical force locally exceeds the surface tension of functional fluid [13]. There are different spraying modes with various E-Jet structures and breaking mechanisms, depending on the applied pressure, flow rate, applied voltages, liquid properties and needle configuration [7]. As a result, E-Jet printing has various limitations and drawbacks that should be addressed. For example, at a low pressure, E-Jet printing has poor print rates that can interfere with micro-structure resolution. Material is consumed or lost during cone-jet disruptions, and the air stream surrounding the interface becomes unstable. There are difficulties when printing droplets of the same size at high pressure and increasing the coffee stain effect.

To date, many studies have presented different high-resolution linear patterns and DoD micro-structures on insulating substrates. Xu et al. emphasized the meniscus dynamics and jetting properties of the E-Jet printing technique. The research was applied to overcome needle obstruction problems during the pulsing drop regime [14]. In a review employing the E-Jet 3D printing technology, Wu et al. sought to produce alternative scaffolds for several specific biological applications [15]. Zou et al. created micro/nanopatterns on polymer substrates by combining E-Jet printing with a wet metal etching method. In the realm of printed electronics fabrication technology, this process is regarded as simple and effective [16]. Liu et al. investigated the droplet trajectory caused by different factors, such as needle size and needle angle [17]. Yang et al. studied E-Jet printing under the influence of various parameters, such as voltage, flow rate and print distance, in a simulation for fabrication micro-structures [18]. Wang et al. simulated and printed micro-drops on the polymer substrate using E-Jet printing to reduce the coffee ring effect under specific conditions [19]. The micro-structures are printed using E-Jet technology by generating a stable cone-jet mode [14,15] and the micro-dripping mode [16,17,18,19]. In both cases, the charged liquid cone-jet strikes the substrate surface to form a linear line before the jet disintegrates into a multi-jet shape [5]. Similarly, the micro-structures produced using the continuous cone-jet formation only form continuous lines and micro-droplets because the ejected ink cone-jet is continuously deposited on the substrate surface [20]. The micro-dripping jet is reported to produce micro-droplets using the DoD E-Jet method. The micro-dripping mode occurs when the pulse voltage is applied [21], or a lower DC voltage is used [17]. Similarly, the modulated AC-pulse was used to print micro-scale droplets on flexible insulating substrates by deploying the E-Jet printing technique. Hence, the silver lines and droplets were printed on the insulating substrate, which has a diameter of 20 µm [22]. In our previous work, Abbas et al. performed a numerical simulation of E-Jet and printing micro-droplets on a flexible substrate. The study calculated the optimized parameters for the development of a DoD E-Jet to print stable micro-structures on a PET substrate [2]. In another study, Abbas et al. introduced a numerical simulation of stable cone-jet formation [23] and printed direct writing structures on flexible substrate. The study offered a 2-phase-field method to optimize parameters for stable cone-jet morphology on a PET substrate utilizing a set of copper control electrodes [24,25,26]. Subsequently, to the best of our knowledge, there are a few research studies in the literature on the variation in droplet fabrication in terms of stability and consistency printed on the polymer substrates by regulating pulse voltage. The use of the combined needle is also limited to discussing cone-jet and micro-drip regimes for printing on PET substrates. In addition, any visual results on controlling the micro-structures’ size and the resolution of the printed droplets and linear patterns still face challenges when using the E-Jet printing technology due to the unfavourable properties of insulating materials [27,28,29]. 

The objective of this research is to use different functional fluids to regulate and sense the bulging and coffee stain effect in multifarious micro-structures produced on a flexible PET substrate. The goal is to introduce a new development in E-Jet printing process and to fabricate micro-patterns that can be used for the application of MEMS devices. After a series of tests, the simulation was run to obtain previously unheard-of values. Within the cone-jet and micro-drip regimes, the resolution and diameters of the printed linear and microscopic patterns were regulated. The experimental work analyses and illustrates the properties and overhead projection display of the printed micro-structures under the constant variation in DC pulse voltages during a periodic pulsed cone-jet regime by correlating these factors (e.g., projection display in microstructures) with the simulation results. As a result, the printed micro-structures are considered essential to be used in the application of different M/NEMS devices by employing E-Jet printing technology. 

## 2. Materials and Methods

### 2.1. Simulation of Electrohydrodynamic Jet

**Figure 1:**
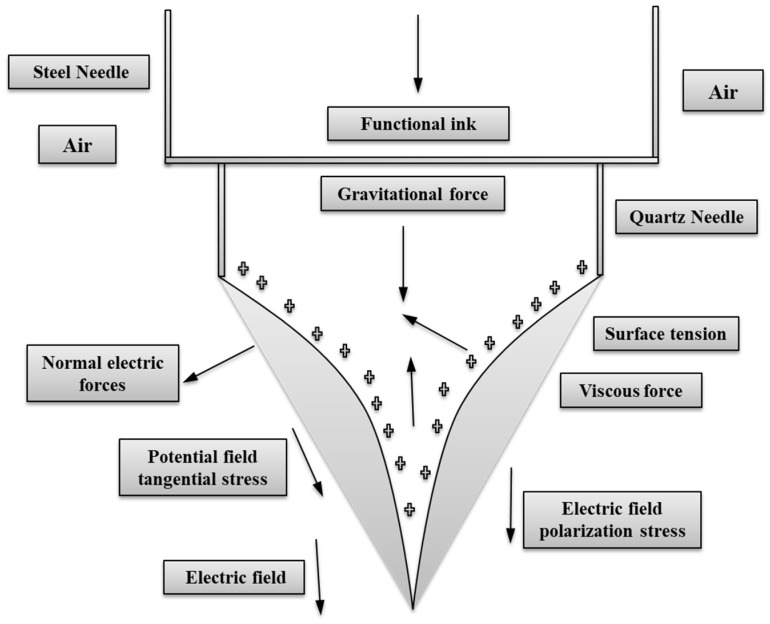
Forces acting around Taylor cone in the E-Jet printing process.

In this simulation investigation, the phase field approach was used to generate stable cone-jet and micro-drip regimes. The phase field approach was exploited to determine the cone-jet’s morphological properties. As a result, many types of forces, such as viscous force, gravitational force, surface tension, and electrical force, play a critical role in the stability of the cone-jet regime. The phase field simulation has already shown that E-Jet printing factors, such as flow rate, pulse frequency, and applied voltage, influence the cone-jet shape and droplet sizes [12]. The phase field approach is preferred in this study to control the influence of different functional fluids on the creation of stable cone-jet and micro-drip regimes in the quest to obtain flexible micro-fabrication on polymer substrates such as PET. During the simulation process and under the influence of electric and hydrodynamic fields, the conservation of mass and Navier–Stokes equations are solved. Then the electrical shearing force and the electrical field jointly induced high viscous shearing force and internal pressure on the inner functional ink. Figure 1 shows a schematic depiction of the distribution forces operating around the needle interface. The simulation is employed in a variety of phases, including fluid and air, which are believed to be incompressible and immiscible [19]. Equation (1) expresses the phases of functional ink.

(1) $${\nabla .u}_{ij}=0$$

where *u_ij_* denotes two distinct functional ink flow rates.

The Taylor–Melcher leaky dielectric model underpins the phase field approach. At the needle apex, the influence of different forces was detected. The Navier–Stokes equation, which is provided in Equation (2), was used to construct the balance of boundary conditions such as electrostatic and hydrodynamic fields [30].

(2) $$\rho \frac{\partial \vec{u}}{\partial t}.(\vec{u}.\nabla ).\vec{u}=\nabla p.+F_{\mathrm{st}}+F_{\mathrm{es}}+F_{v}+F_{g}+F_{i}$$

where F_st_, F_es_, F_v_, F_g_, and F_i_ are the surface tension, electrical force, volume force, gravity force, and pressure force created by inner functional ink, respectively. As a result, a continuity equation representing a driving force larger than zero was solved during the phase field approach to construct the interface between the liquid and air phases. Equation (1) shows the equations for the combined needle interface (3) and (4).

(3) $$\begin{array}{l} \frac{\partial \phi_{i}}{\partial t}-\nabla .\vec{\mu }\phi_{i}=(\nabla .\frac{\gamma \lambda }{\sum_{T}}).\Delta \psi ,\phi =phipf\\ \lambda =\frac{3\varepsilon phi\sigma }{\sqrt{8}},\gamma =Χ \end{array}$$

(4) $$\begin{array}{l} \left\{ \begin{array}{l} \frac{\partial \phi_{i}}{\partial t}-\nabla .\vec{\mu }\phi_{i}=(\nabla .\frac{{Μ}_{o}}{\sum_{T}}).\Delta \phi_{i}\\ \phi_{i}=\frac{2\sum_{T}}{\theta }\sum_{i}\prod i\mp j-[\frac{1}{\sum_{j}}(0\leq \phi \leq 1)](\frac{1}{2}-2\varepsilon c\Delta \theta \psi ) \end{array}\right.\\ \phi_{A}=phiA,\phi_{B}=phiB \end{array}$$

where ϕ describes the transition state phase field variables based on Cahn–Hilliard equations [31]. The electric field is created by establishing the electrostatic charge physics surrounding the needle outlet wall, which results in body forces, as shown in Equation (5)

(5) $${\vec{F}}_{es}=q\vec{E}-\frac{1}{2}E^{2}\varepsilon +\nabla G\varepsilon_{0}$$

where G is a driving force, which is also known as chemical potential and given in Equation (6)

(6) $$G=\lambda \left[-(\nabla^{2}.\varphi \varphi +\frac{\varphi .(\varphi -1)}{\theta^{2}})\right]=\frac{\delta \lambda }{{\delta \theta }^{2}}(\psi \varphi )$$

where λ is the combined energy density of the two separate functional fluids, and similarly δ is the width of a metal needle, also known as an interface thickness. The contact angle $\theta^{2}$ between the polymer substrate and the functional ink is determined by computing the body forces.

### 2.2. Optimized Micro-Fabrication Design Considerations

**Figure 2:**
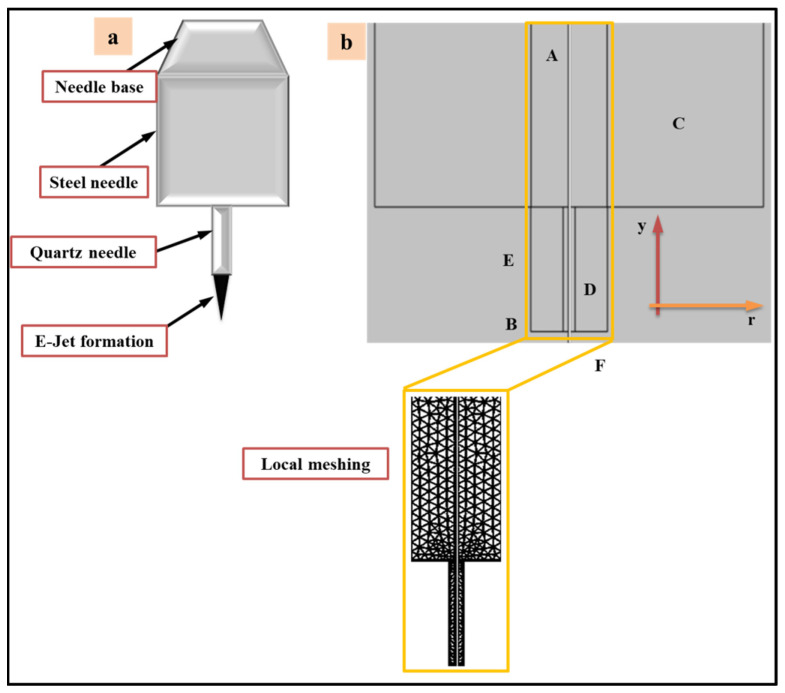
The numerical simulation of E-Jet printing (**a**) combined needle structure (**b**) The geometric model and basic boundary conditions and the local refined meshing scheme.

COMSOL multi-physics software was used to simulate the cone-jet and micro-drip regimes. The combined needle system was created to validate the experimental design for the micro-fabrication process. The 2D axisymmetric drawing was primarily generated to build an accurate model to pursue suitable simulation results, as illustrated in Figure 2. The geometry of the combined needle created in this simulation model is shown in Figure 2a. Similarly, Figure 2b depicts the model’s boundary conditions and tighter, user-controlled meshing, which can have a considerable influence on simulation fidelity. To execute the E-Jet printing model in the phase field domain, an axisymmetric geometry was created. As can be observed, the cone-jet regime in E-Jet technology involves significant electrostatic physics, which influence the charge density distribution around the combined needle. The findings were achieved at the optimal settings, where a series of simulation tests were carried out to determine the appropriate parameters for two distinct functional inks of UF 3808 and SEMICOSIL988/1K. The simulation study focuses on the regime transitions at different time intervals of a two-phase interface. Table 1 assumes and defines the boundary conditions of the electric charge field and the two fluid fields. Furthermore, the functional restrictions of liquid interfacial tension and air pressure applied to the steel needle during the cone-jet production phase had a significant influence, lowering the diameter of the jet to become even smaller than the inner diameter of a needle.

**Figure 3:**
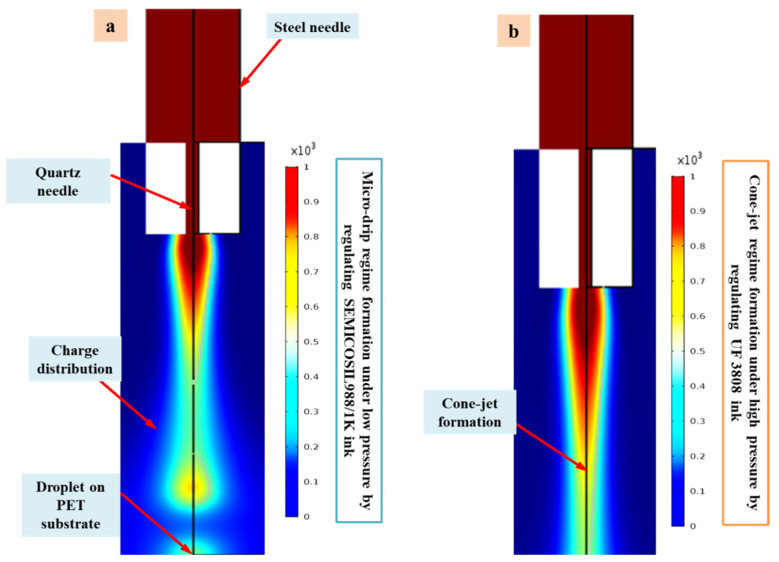
(**a**) Photograph of specific simulated outcomes obtained under optimum settings during the micro-drip regime (**b**) image of specific simulated results obtained with optimal settings during the cone-jet regime.

A series of simulation tests were carried out in parallel using two distinct functional inks to establish the optimal printing parameters of multidimensional micro-structures. Then, during the micro-drip regime, the applied pressure of 40 kPa, DC high-pulse voltage of 1.95 kV, duty cycle of 80%, pulse frequency of 60 Hz, printing height of 0.25 mm, and printing speed of 1 mm/s were chosen as optimum parameters. During the simulation, the functional ink of SEMICOSIL988/1K was employed to establish a micro-drip regime and unique parameters were found. Following that, we ran a series of simulations with UF 3808 as the functional ink to produce the cone-jet regime. We discovered this by holding the entire parameters constant and raising the applied pressure to 50 kPa at 1.60 kV. This shows that utilising the value of the applied dc voltage may continually maintain the cone-jet regime and then generate the suitable Taylor cone form. The flexible micro-structures were then directly printed on the polymer PET substrate during the experimental investigation. The phase field model created in this study is a helpful tool for studying the E-Jet printing process on polymer substrates. The E-Jet approach is a potential way of creating micro-scale structures for M/NEMS equipment. Figure 3 depicts the specific simulated outcomes under the improved settings for the cone-jet and micro-drip regimes.

### 2.3. Experimental Details

**Figure 4:**
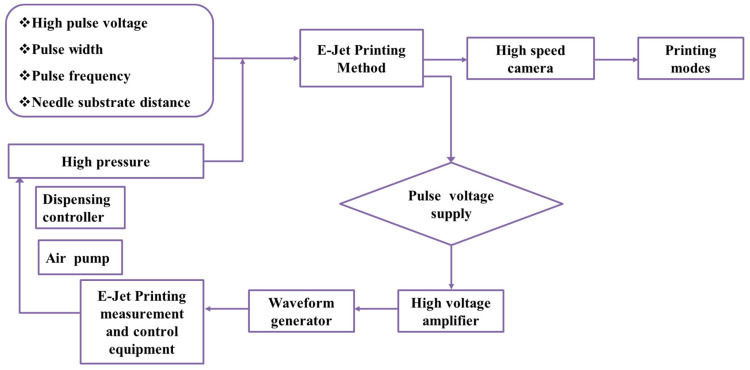
Flow diagram of the E-Jet printing process with pulse voltage implementation.

The experimental setup was established in an existing study based on E-Jet printing technology, which consists of a combined needle system (steel and quartz), an air pump dispensing controller (PHD ULTRATM, Harvard Appsratus, Holliston, MA, USA), a DC pulse power supply, a computer-controlled X-Y-Z movement station and a microscopic vision system [2]. The inner and outer diameters of the steel needle were 365 μm and 700 μm, respectively. Similarly, the inner and outer diameters of the quartz needle were maintained at 50 μm and 365 μm, respectively. The distance between the combined needle and the substrate was maintained at 1 mm. Similarly, a flow diagram of the E-Jet printing process with pulse voltage implementation is shown in Figure 4. The combined needle system was connected to a DC positive pulse voltage power supply and a further inlet of needle base was connected to the air pump.

**Figure 5:**
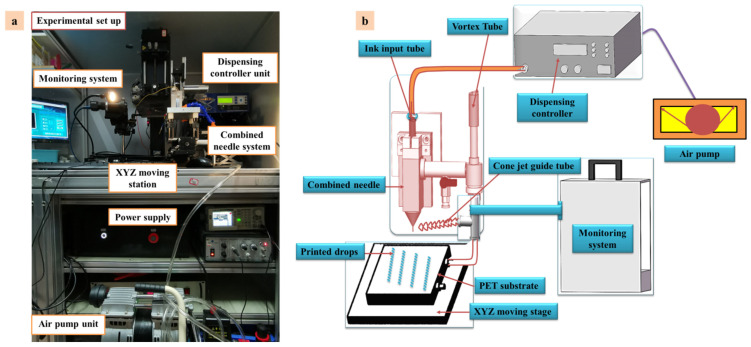
The (**a**) experimental setup (**b**) schematic diagram of the E-Jet printing process.

In this work, two different functional inks of UF 3808 and SEMICOSIL988/1K were used at the semiconductor packaging level to explore the influence of various process parameters and stability of the cone-jet on PET substrate through experiments. The physical properties of functional inks (Henkel Corporations Group Ltd., Stamford, CT, USA) were achieved by the suppliers. However, this material can provide excellent mechanical properties for electronic devices, and is widely used in flexible printed electronics. It is easily cured by light. The dynamic viscosity of the used inks is 450,000 cps. They have excellent bonding strength and resistance to heat and humidity cycles, and can provide a robust strength to insulating substrates. The physical performance parameters of the functional inks are given in Table 2. We aimed to print stable micro-structures on the flexible insulating substrates (i.e., PET), which were 0.5 mm and 1 mm thick. The DC positive pulse voltage power supply was used to create a considerable electric field between the steel needle and ground electrode at constant parameters. The air pump was used to provide a hydrodynamic force to push functional liquid towards the needle outlet. Voltage signals were produced using a positive-polarity, high-voltage amplifier (Smart Material GmbH, Löbtauer Str., Dresden, Germany), which was connected to a function generator (FG-7002C, EZ digital, Binh Thanh District, Ho Chi Minh City, Vietnam), and were monitored using a high-speed camera (Fastcam SA4, Photron, Westfield, MA, USA). The peak in the dc pulse voltage was 1.95 kV and pulse frequency was 1000 Hz. The duration of pulse frequency was set as a half of a period. The substrate was connected to a ground electrode power supply. The substrate was moved at a speed of 100 mm/s to a computer-controlled moving station. The schematic and experimental E-Jet printing setup used to generate a stable cone-jet is shown in Figure 5.

## 3. Results and Discussion

### Stable Micro-Patterns on Flexible Substrate

**Figure 6:**
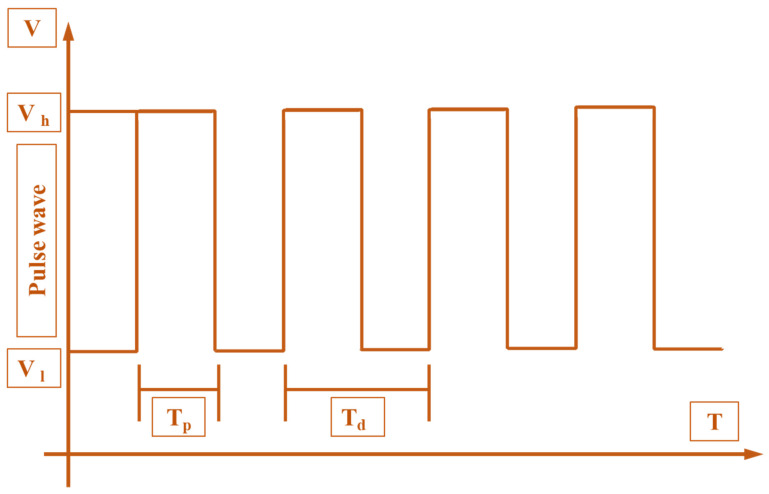
Schematic diagram of pulse waveform in terms of voltage across time duration.

The DC positive pulse voltage was selected and periodic electric field force was applied to control the formation and interruption of the E-Jet flow, to realize the DoD printing of droplet patterns on the flexible substrate. Figure 6 is a schematic diagram of the pulse waveform, where Vh represents the high-voltage pulse, Vl represents the low-voltage pulse, and the difference between Vh and Vl represents the voltage amplitude. In one cycle, the ratio of the bandwidth Tp of a high-voltage pulse to the total period Td of the pulse voltage is called a duty cycle. Further, the reciprocal of a pulse voltage period, Td, is called the pulse frequency *f*, which represents the number of times the high-voltage pulse signal is generated per unit time. Similarly, it directly disturbs the resolution and density of the micro-structures formed during the entire process. Among them, the choice of a high-voltage pulse should ensure that the end of the spray needle produces a stable micro-dripping mode or cone jet mode. The choice of a low-pressure pulse should ensure that the end of the spray needle can form a Taylor cone profile but does not produce a cone-jet. The use of pulsed electrical signals can reduce the charge of the flexible insulating substrate. Therefore, the effect of residual charges on the stability of the cone-jet and micro-droplets are considerably reduced.

**Figure 7:**
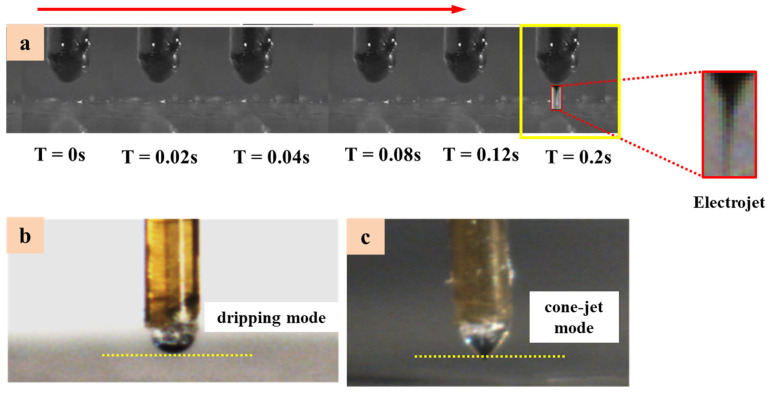
The (**a**) formation and evolution of electrojet in space to print on PET substrate by increasing time intervals (**b**) micro-dripping regime when no voltage at needle (**c**) cone-jet regime.

A series of experiments were performed using a 0.5-mm and 1-mm-thick PET substrate and a stainless-steel quartz nozzle with an inner diameter of 50 μm. The applied pressure, high-voltage pulse, low-voltage pulse, pulse duty cycle, pulse frequency, printing height and printing speed (i.e., 40 kPa, 1.95 kV, 1.60 kV, 80%, 60 Hz, 0.25 mm and 1 mm/s) are obtained as optimized parameters for printing stable micro-structures. The formation and evolution of the electrojet on a PET substrate, achieved by increasing the different time intervals (i.e., 0 s, 0.02 s, 0.04 s, 0.08 s, 0.12 s and 0.2 s), are shown in Figure 7a. Figure 7b is the functional ink form dripping mode when no voltage is applied and Figure 7c is the cone-jet form under a high pulsed voltage. It can be seen from Figure 7a that the printing cycle of a single droplet is 0.2 s. When T = 0 s, the functional ink at the end of the quartz needle is hemispherical and begins to evolve into a conical morphology. When T = 0.12 s, the Taylor cone forms and merges, which produces cone-jetting phenomena. When T = 0.2 s, the cone-jetting is completed and the functional ink returns to a hemispherical shape. 

**Figure 8:**
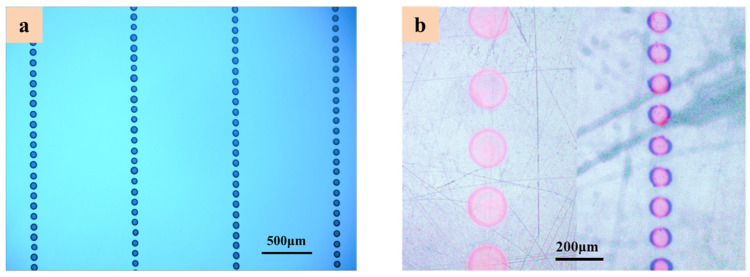
The DoD E-Jet printing of micro-droplets on flexible PET substrates (**a**,**b**) using functional liquid of SEMICOSIL988/1K.

This work uses SEMICOSIL988/1K and UF3808, two different types of functional inks, to print printing micro-droplets and linear patterns on the flexible substrates. The optimized process parameters are properly adjusted to integrate the electrofluid jet for the stable droplets’ morphologies. The printing control monitoring camera performs linkage control for various parameters, such as motion, pressure, voltages, and flexible insulating substrates. The micro-droplets of different sizes were printed on the flexible PET substrates with the execution of DoD E-Jet printing process as shown in Figure 8. Moreover, Figure 8a displays the different variations of several droplets in terms of their sizes that were printed in the form of array using functional ink of SEMICOSIL988/1K. The minimum droplet diameter was measured at 104 μm on the PET substrate surface. Similarly, Figure 8b shows micro-droplets being printed and the droplet diameter was measured at 85 μm. The DoD printing of droplets shows that the size of droplet is not irregular, which leads to high quality and efficient printing during the production of overhanging structures with a specific ink. The droplet morphology allows for the suppression of the adhesive flexible structure in the electronic packaging process, which avoids the coffee stain effect on droplet morphology. Due to this phenomenon, the droplet morphology does not contradict the highly insulating properties of a PET substrate and the formed structure is orderly and controllable.

**Figure 9:**
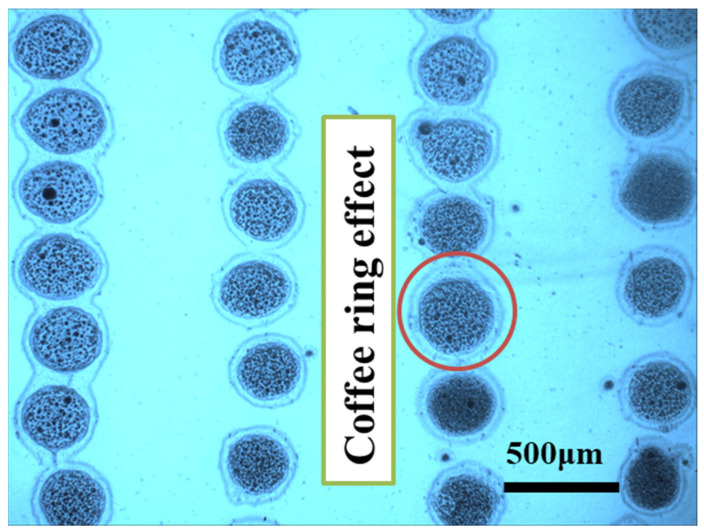
The unstable printed drops under 40 kPa pressure using functional liquid of SEMICOSIL988/1K.

When the applied pressure is increased to more than 50 kPa in the case of SEMICOSIL988/1K ink, the printed drop size rises. The research reveals that each individual droplet is not uninterrupted and implying that the interior form of the drop is not continuous due to the coffee ring effect, which is easily discernible. However, there is no visible occurrence of the coffee ring effect below 40 kPa, suggesting that the ink flow particle size is different. This generates various drop forms. In the future, we hope to increase the uniformity of the printed droplets, as well as the discontinuous behaviour in micro-structures. This will require plasma treatment procedures that are often used to increase the surface energy of polymers such as polypropylene (PP) and PET. Therefore, Figure 9 depicts many changes in the size of printed droplets due to the oscillation in their shapes. It also happens because of different printing settings for the experiments have different influence of process parameters. It also focused exclusively on the functional ink properties and then measured the minimum droplet size of 305 μm on the substrate surface. 

**Figure 10:**
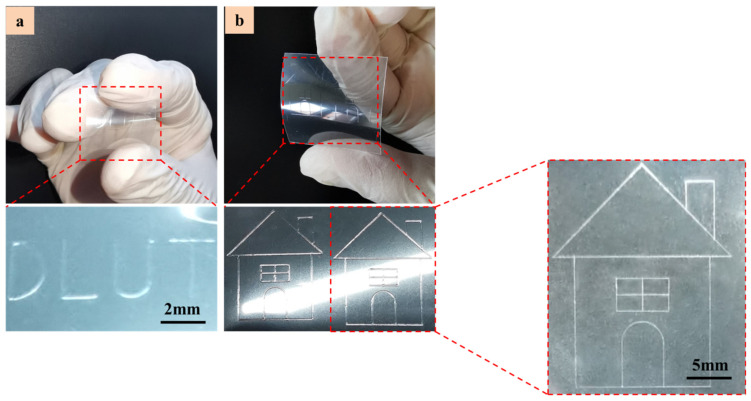
The printing of linear patterns on flexible PET substrates using functional liquid of UF3808 (**a**) “DLUT” pattern (**b**) “Little house” pattern.

Furthermore, Figure 10 shows the different linear patterns printed on a flexible PET substrate with a thickness of 1 mm. The applied pressure of 50 kPa was used to increase the resolution and consistency of the printed micro-structures while keeping all process parameters constant. During the printing process, parameters such as printing trajectory, printing speed, voltage and pressure were precisely linked and controlled. Figure 10a,b display the “DLUT” and “small house” patterns printed at room temperature using UF 3808 with a viscosity of 450,000 cps. The structure is uniform and continuous, and there is no glue line breakage and accumulation. The structure line space is about 200 μm between the letters, and the aspect ratio is about 0.4. The printed micro-structures indicate that the smallest feature dimension (line width) in these patterns is about 500 μm. These simple continuous linear features display that the carried charge in droplets is neutralized on these highly insulating substrates using DC pulse voltages. Otherwise, the accumulated residue charge and the resulting repulsion between droplets make continuous printing almost impossible.

## 4. Conclusions

In this paper, we used the electro-fluid jet printing platform and referred to the analysis of the simulation and experiment results. We carried out research on the influence of electrojet printing regimes, e.g., cone-jet and micro-drip, for micro-patterns on a polymer PET substrate. The influence of process parameters such as DC pulse voltage and pulse frequency on the size and morphology of the formed micro-structure indicates that the size of a micro-droplet is directly proportional to the pressure. Likewise, it is inversely proportional to the printing speed and high-pressure pulse. Primarily, the droplet size decreases and then increases with the increasing printing height. Subsequently, the influence of voltage, pressure, printing speed and printing height on the electrojet printing of flexible micro-fabrication was studied. The printing process conditions were obtained from the simulations to observe the size and shape of the micro-patterns’ morphologies. By generating advantageous cone-jet and micro-drip regimes, it showed that the bulging and coffee stain effect was not predominant on a flexible PET substrate with the regulation of two different functional fluids. Finally, based on the range of influencing parameters, e.g., a metal-quartz spraying needle, functional ink viscosity and the surface energy of polymer substrate, the printed results are stable and versatile. The printing results are useful and can be applied to different polymer selections. The experimental design is deemed to be easier to implement, cost-effective, and tailored to polymer sectors. It has been concluded and demonstrated in this study that the E-Jet printing technology is a suitable method for patterning stable micro-structures on the polymer substrates. More, the polymer substrates are currently considered the most compatible material for application of biomedical devices and M/NEMS devices. 

## Figures and Tables

**Table 1:** The boundary conditions for electric charge field and various fluid fields.

Boundary Condition	Electric Charge Field	Fluid Field
A: Steel needle inlet	***ϕ*** = V_0_	u = Q_metal_/A_metal_
B: Quartz needle inlet	***ϕ*** = V_0_	u = Q_quartz_/A_quartz_
C: Wall of steel needle	***ϕ*** = V_0_	u = 0
D: Wall of quartz needle	***ϕ*** = V_0_	u = 0
E: Axisymmetric model	***ϕr*** = 0	u_r_ = 0
F: Outlet of needle	***ϕ*** = 0	*p* = 0

**Table 2:** Physical characteristics of the different functional inks used in existing work.

Functional Ink	Density (g·cm^−3^)	Dynamic Viscosity (m⋅Pa·s)	Surface Tension (N/m)	Storage Modulus (N/mm^2^)	Dielectric Constant
UF 3808	1.16	450,000	0.031	260	3.24
SEMICOSIL988/1K	1.1	450,000	0.045	350	2.38

## Data Availability

Not applicable.
